# Supplementary material for: Content-rich biological network constructed by mining PubMed abstracts
Source: BMC Bioinformatics. 2004 Oct 8;5:147. doi: 10.1186/1471-2105-5-147 (PMC528731; doi:10.1186/1471-2105-5-147)
Supplement: Additional File 5 — The original Chilibot query results of the term "long-term potentiation (LTP)" and 22 other terms, limiting the latest references analyzed to the years 1990, 1995, 2000, and 2004. [file 1471-2105-5-147-S5.bz2 › chilibotAdditionalFile5/ltp2000/html/CREB_TAU.html]

 


 **CREB** and **TAU** 
  
Found 10 abstracts in PubMed,  **10 abstracts were retrieved and analyzed**.  


---

 Search Google  |
 PDF files only 
|  EDU domain only 

---

**Interactive relationship** (e.g. stimulation, inhibition, etc)

- Screening of putative target proteins showed that the  **tau**  1 core can interact with the C terminal part of the  **CREB**  binding protein CBP.  Ref: 9649342 Biochemistry, 1998

**Parallel relationship** (e.g. studied together, co-existance, homology, etc.)

- Elevated ICER levels in decidualized ES cells may be indicative of the presence of overriding amounts of transcriptional activators such as full length CREM  **tau**  or  **CREB**  which, in turn, upon cAMP induced phosphorylation, contribute to the induction of the dPRL gene.  Ref: 8994192 Mol Endocrinol, 1997
- It is present in the nucleus of the cells in which it is expressed and can phosphorylate and activate the cyclic AMP response element binding proteins  **CREB**  and CREM  **tau**  in a manner analogous to protein kinase A.  Ref: 9238860 Recent Prog Horm Res, 1997
- in contrast, the expression of CREM  **tau**  and  **CREB**  activators is more diffuse and generalized.  Ref: 8386526 Neuron, 1993
